# Characterization of the Kallikrein-Kinin System Post Chemical Neuronal Injury: An *In Vitro* Biochemical and Neuroproteomics Assessment

**DOI:** 10.1371/journal.pone.0128601

**Published:** 2015-06-05

**Authors:** Amaly Nokkari, Tarek H. Mouhieddine, Muhieddine M. Itani, Wassim Abou-Kheir, Georges Daoud, Rui Zhu, Yehia Meshref, Jihane Soueid, Moustafa Al Hariri, Stefania Mondello, Ayad A. Jaffa, Firas Kobeissy

**Affiliations:** 1 Faculty of Medicine, Department of Biochemistry and Molecular Genetics, American University of Beirut, Beirut, Lebanon; 2 Faculty of Medicine, American University of Beirut Medical Center, Beirut, Lebanon; 3 Faculty of Medicine, Saint George University of London, Nicosia, Cyprus; 4 Faculty of Medicine, Department of Anatomy, Cell Biology and Physiology, American University of Beirut, Beirut, Lebanon; 5 Department of Chemistry and Biochemistry, Texas Tech University, Lubbock, Texas, United States of America; 6 University of Messina, Department of Neurosciences, Messina, Italy; Bristol Heart Institute, University of Bristol, UNITED KINGDOM

## Abstract

Traumatic Brain Injury (TBI) is the result of a mechanical impact on the brain provoking mild, moderate or severe symptoms. It is acknowledged that TBI leads to apoptotic and necrotic cell death; however, the exact mechanism by which brain trauma leads to neural injury is not fully elucidated. Some studies have highlighted the pivotal role of the Kallikrein-Kinin System (KKS) in brain trauma but the results are still controversial and inconclusive. In this study, we investigated both the expression and the role of Bradykinin 1 and 2 receptors (B1R and B2R), in mediating neuronal injury under chemical neurotoxicity paradigm in PC12 cell lines. The neuronal cell line PC12 was treated with the apoptotic drug Staurosporine (STS) to induce cell death. Intracellular calcium release was evaluated by Fluo 4-AM staining and showed that inhibition of the B2R prevented calcium release following STS treatment. Differential analyses utilizing immunofluorescence, Western blot and Real-time Polymerase Chain Reaction revealed an upregulation of both bradykinin receptors occurring at 3h and 12h post-STS treatment, but with a higher induction of B2R compared to B1R. This implies that STS-mediated apoptosis in PC12 cells is mainly conducted through B2R and partly via B1R. Finally, a neuroproteomics approach was conducted to find relevant proteins associated to STS and KKS in PC12 cells. Neuroproteomics results confirmed the presence of an inflammatory response leading to cell death during apoptosis-mediated STS treatment; however, a “survival” capacity was shown following inhibition of B2R coupled with STS treatment. Our data suggest that B2R is a key player in the inflammatory pathway following STS-mediated apoptosis in PC12 cells and its inhibition may represent a potential therapeutic tool in TBI.

## Introduction

Traumatic Brain Injury (TBI) represents a major public health concern, as it affects a wide number of the population. According to the Centers for Disease Control and Prevention, 1.7 million people sustain TBI in the United States each year, among which, 52,000 die [1]. Although the prevalence of brain trauma is the highest in warzones, with a total of 307, 287 TBI cases in the U.S. army since 2000 [2], TBI is also seen in car accidents, sport injuries [3] and falls, especially among the elderly [4]. Brain injury involves a primary and a secondary phase. The primary injury results immediately from the initial head trauma and is followed by or intertwined with secondary injury events [5]. Neuro-inflammation is among the main secondary injury mechanisms following TBI and it represents a potential target for therapeutic intervention [6]. It is known that TBI provokes apoptotic and necrotic cell death through the activation of the protease system, namely the calpain/caspase system [7]. However, the exact mechanism through which brain trauma leads to neuronal injury remains to be identified.

Of interest, the vasodilator Kallikrein-Kinin System (KKS) represents one of the major inflammatory pathway activated following tissue damage [8]. The main role of the KKS is to release pro-inflammatory kinins that react on the cell through either the inducible Bradykinin 1 receptor (B1R) or the constitutive Bradykinin 2 receptor (B2R) [9]. Although this important vasodilator system has been well characterized in the areas of diabetic nephropathy [10], diabetic retinopathy [11] and cardiovascular diseases [12]; its role in brain injury remains highly controversial [13]. Indeed, a study by Albert-Weissenberger et al. states that inhibition of the B2R offers no protection following a closed head model of focal TBI in mice, even 7 days post-TBI, in contrast to inhibition of the B1R, which improves neurological outcome after focal closed head injury by reducing axonal damage and astroglia activation [13]. Nevertheless, another study by Hellal et al. confirms that inhibition of the B2R with the antagonist LF 16–0687 reduced neurological deficit and cerebral edema 4 hours post-closed head trauma in mice [14]. Similarly, Trabold et al. worked on B2R knockout mouse model and showed that B2R and bradykinin (Bdk) are involved in brain edema formation and cell death after TBI [15].

Moreover, the use of *in vitro* models has been proposed in the area of KKS [16–19]. Among these models, PC12 cell line has been utilized as an *in vitro* model to mimic *in vivo* studies including neuronal apoptosis, necrosis and autophagy [20–24]. Ballesteros et al (2007) and Minambres et al (2008) demonstrated a direct association between the presence of apoptosis-related proteins in TBI patients and the apoptotic effects of jugular bulb sera from patients with TBI on neuronal cells PC12 [25, 26]. There was also a direct correlation between apoptotic rate in PC12 cells and TBI patient outcome after 6 months [25]. Thus, PC12 cells were considered a valid *in vitro* neuronal model to study neural injury mechanisms as depicted in TBI.

Furthermore, it is classically agreed upon that severe calcium dysregulation promotes necrotic cell death. Nevertheless, new studies suggest a link between apoptosis and calcium [27]. It is stated that controlled intracellular calcium increase, induced by mild chemical insult, provokes cell death through apoptosis [28].

Therefore, this manuscript aims at better understanding the mechanism of Bdk-mediated neuronal injury in PC12 cells upon exposure to the apoptotic-neurotoxic drug Staurosporine (STS), as well as the role of calcium in this mechanism. In addition to the classical biochemical testing, a global neuroproteomic analysis was also conducted to assess the implicated altered pathways involved post-STS treatment.

## Materials and Methods

### Cell culture and Treatments

PC12 cells (PC-12 ATCC CRL-1721) were cultured and maintained in Dulbecco’s Modified Eagle Media (Lonza) supplemented with 10% heat inactivated fetal bovine serum (Sigma), 5% heat inactivated horse serum (Sigma) and 1% Penicillin/Streptomycin (Sigma). Cells were incubated at 37°C in a humidified incubator (5% CO_2_). PC12 cells were pretreated for 1h with either: 0.1 μM [Hyp^3^]-Bradykinin (*B7775*, Sigma); 1 μM HOE-140 (*BML-NK104-0001*, Enzo Life Sciences); 0.1 μM Bradykinin Fragment 1–8 acetate salt hydrate (*B4397*, Sigma); 1 μM R-715 TFA salt (*R9032*, Sigma). All pre-treatments were reconstituted in dH_2_O. Following pre-treatment, PC12 cells were treated for 3h, 12h and 24h with Staurosporine 1 μM (*569397-100UG*, Calbiochem), reconstituted in DMSO.

### Immunofluorescence and Confocal Microscopy

PC12 cells were seeded on glass cover slips in 12-well plates (150x10^3^ cells). Treated cells were fixed with 4% formaldehyde for 10 minutes, followed by permeabilization with 0.5% Triton X-100 for 3 minutes and then nonspecific sites were blocked by incubating cells with 1X PBS containing 2% BSA for 30 minutes. Cells were then probed with either B2R (Santa Cruz sc-25671, polyclonal rabbit) or B1R (Santa Cruz sc-25484, polyclonal rabbit) primary antibodies in blocking solution, and were then incubated overnight at 4^°^C. Cells were washed with PBS containing 0.1% Tween-20, incubated with Alexa-488 and/or 568 conjugated IgG in 2% BSA for 1 hr at room temperature, and finally washed and mounted using the antifade reagent Fluoro-gel II with DAPI. Fluorescent signals were captured using Zeiss LSM 710 confocal microscope and images were acquired and analyzed using the ZEN image software.

### Fluo-4 AM Calcium Staining

PC12 cells were seeded on glass cover slips in 12-well plates (150x10^3^ cells). Treated cells were incubated with 2 μM Fluo-4 AM (*F-14201*, Molecular Probes Life Technologies) for 15min in the dark. Cells were then washed with PBS and glass cover slips were mounted using the antifade reagent Fluoro-gel II with DAPI. Fluorescent signals were captured using Zeiss LSM 710 confocal microscope and images were acquired and analyzed using the ZEN image software.

### Real-time Polymerase Chain Reaction (RT-PCR)

PC12 cells were seeded in petri dishes (2.5x10^6^ cells) and STS-treated for 3h and 12h. RNA extraction was performed using the GenElute Mammalian Total RNA Miniprep Kit (Sigma). Subsequently, RNA content was quantified using a spectrophometer (nanodrop ND-1000). cDNA was synthesized from 1 μg of total cellular RNA using the RevertAid First Strand cDNA Synthesis Kit (Thermo Scientific). Real-time PCR was then performed using a 2x RT-PCR Master Mix in a CFX96 system (Bio-Rad) with specific primers for B1R (F: GCGACGGCAAGCCCAAGCTA; R: TGCCAAGCCTCGTGGGGGAA) and B2R (F: GCTTGGCGTGCTGTCGGGAT; R: TCGGAAGCGCTTGCCCACAA) genes (TIB Molbiol). The reaction was performed as follows: precycle of 95°C for 10 minutes, followed by 40 cycles each consisting of an initial 95°C step for 10 seconds, then 58°C for 30 seconds, 72°C for 1 minute and finally an extension step consisting of 72°C for 10 minutes. The results were analyzed using the ΔΔCT method.

### PC12 Cells Protein extraction and Quantification

PC12 cells were cultured in petri dishes (2.5x10^6^ cells) and incubated at 37°C, 5% CO_2_. The treated cells were collected by trypsinization. Protein extraction was performed by adding RIPA lysis buffer, along with protease and phosphatase inhibitors (Roche Life Science), and dithiothreitol. Protein quantification was achieved using the DC Protein Assay (Bio-Rad) as per manufacturer’s recommendations employing bovine serum albumin as a standard. Finally, equal amount of proteins re-suspended in lysis buffer and Laemmli buffer 2X (Biorad), were boiled for 5min and stored at -20°C.

### Western Blot Analysis

Samples were loaded into sodium dodecyl sulfate-polyacrylamide gel, subjected to electrophoresis, and transferred onto a methanol activated polyvinylidene fluoride membrane overnight. The membranes were blocked for 1h with 5% fat-free milk prepared in phosphate-buffered saline containing 0.05% Tween-20. Membranes were incubated overnight at 4°C with B2R antibody (Abcam ab101704, rabbit polyclonal, 1:1000) and anti-β Actin (Sigma A2228, mouse monoclonal, 1:1000). Then, membranes were incubated with HRP-conjugated secondary antibody (Santa Cruz) and protein bands were visualized using enhanced chemiluminescence (Roche Life Science).

### Mass Spectrometry Data Analysis

A tryptic digested 1-μg aliquot of each protein sample was subjected to LC-MS/MS analysis. LC-MS/MS was acquired using Dionex 3000 Ultimate nano-LC system (Dionex, Sunnyvale, CA), LTQ Orbitrap Velos and TSQ Vantage mass spectrometers (Thermo Scientific, San Jose, CA). The LC elution gradient of solvent B was: 5% over 10 min, 5%-20% over 55 min, 20–30% over 25 min, 30–50% over 20 min, 50%-80% over 1 min, 80% over 4 min, 80%-5% over 1 min and 5% over 4 min. Solvent B consisted of 100% ACN containing 0.1% formic acid while solvent A composed of 98% HPLC water containing 0.1% FA. The LTQ Orbitrap Velos mass spectrometer was operated in data-dependent acquisition mode comprised of two scan events. The first scan event was a full MS scan of 380–2000 *m/z* at a mass resolution of 15,000. The second scan event was CID MS/MS of parent ions selected from the first scan event with an isolation width of 3.0 *m/z*, using 35% normalized collision energy of 35%, and an activation Q value of 0.250. The CID MS/MS scans were performed on the 10 most intense ions observed in the MS scan event. The dynamic exclusion was set to have repeat count of 2, repeat duration of 30s, exclusion list size 200 and exclusion duration of 90s.

LC-MS/MS raw data was used to generate mascot generic format file (*.mgf) by Proteome Discover version 1.2 software (Thermo Scientific) then searched using SwissProt database (Rattus) in MASCOT version 2.4 (Matrix Science Inc). Iodoacetamide modification of cysteine was set as a fixed modification, while oxidation of methionine was set as a variable modification. An *m/z* tolerance of 6 ppm was set for the identification of peptides with maximum 2 missed cleavages. Also, tandem MS ion tolerance was set to 0.8 Da with label-free quantification. Scaffold Q+ version 3.6 (Proteome Software, Portland, OR) was employed for spectral counts quantitation. Independent t-tests (α = 0.05) were performed on each sample group compared to the control.

### NeuroSystems Biology Analysis

The altered pathways relevant to different treatments were analyzed using Pathway Studio software v.10 (Ariadne Genomics, Rockville, MD, USA). This software helps to interpret biological meaning from gene (protein) expression, build and analyze pathways, and find relationships among genes, proteins, cell processes, and diseases. This software comes with a built-in resource named ResNet, which is a database of molecular interactions based on natural language processing of scientific abstracts in PubMed. Using ResNet, a researcher can simply analyze gene product/protein list and build a pathway using well-known interactions discussed in existing literature. In our study, identified altered protein lists of STS and STS+B2R inhibitor (STS+B2I) treatment were subjected to systems biology analysis for network identification. Differential pathways were generated using the “direct interaction” algorithm to map cellular process and interactions between altered proteins. The program searches the current pathway database and ResNet for interactions with the selected entities, and adds them to the pathway. After the new pathway was built, we were able to obtain more detailed information of the altered putative pathways involved upon STS treatment.

### Statistical Analysis

Protein levels were quantitated using the Image J software. The areas were manually measured with the rectangle tool. Results of all measurements were expressed as mean ± standard error of the mean (SEM). One-way measures analysis of variance (ANOVA) tests were used to assess differences between groups, followed by Bonferroni post-hoc tests. Paired t tests were used to assess differences between B1R and B2R gene expression within experimental groups. All hypothesis tests conducted were 2-tailed and probability values (*p*) less than 0.05 were considered significant. Statistical analysis was performed using the SPSS 21.0 software package (SPSS Inc, Chicago, Illinois, USA)

## Results

### B2R is highly expressed in PC12 cells post-STS treatment

**Fig 1:**
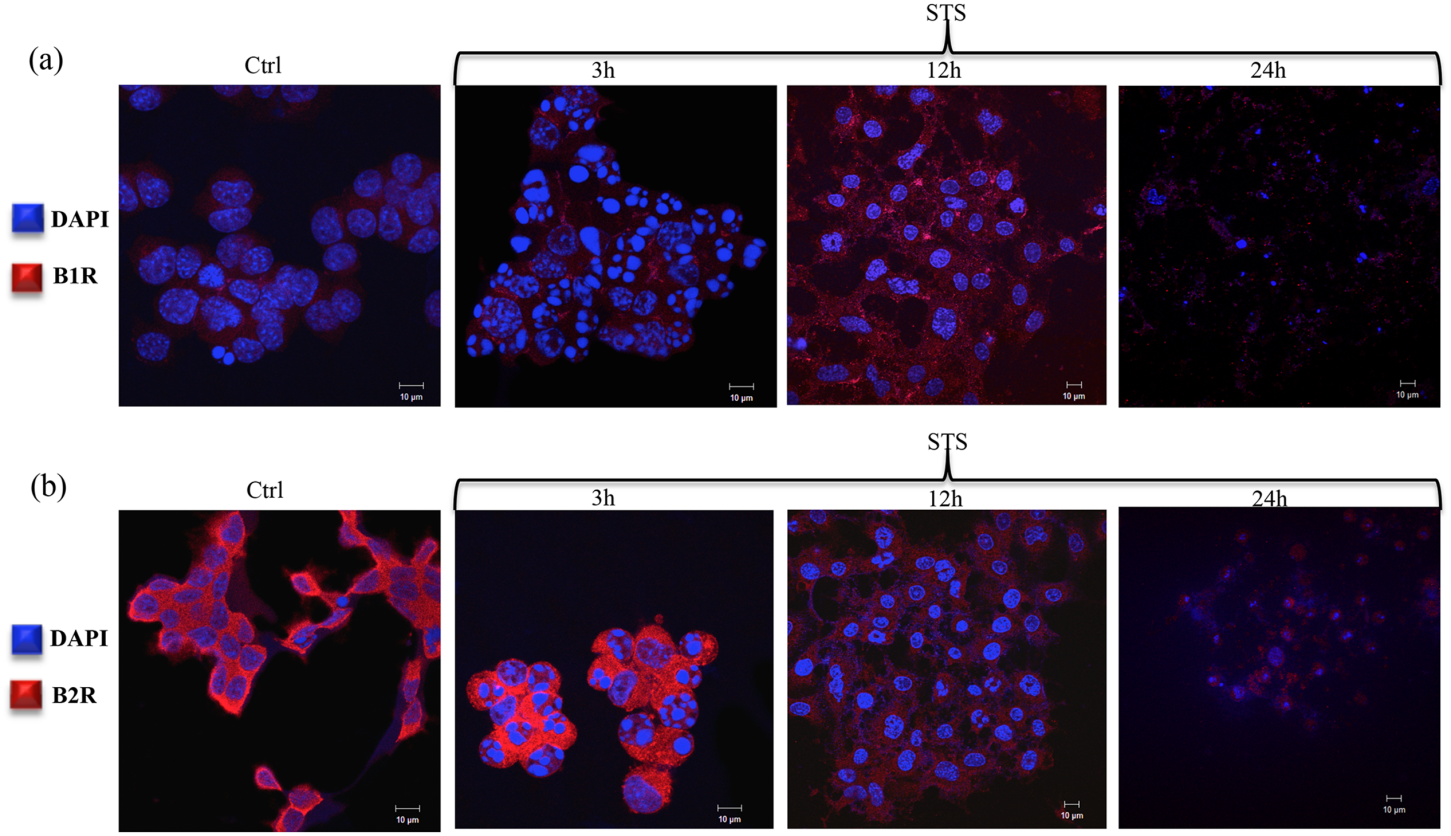
Immunofluorescent analysis of Bradykinin 1 and 2 receptors expression in PC12 cells. Immunofluorescent images of **(a)** B1R and **(b)** B2R in PC12 cells in response to 3h, 12h and 24h of STS treatment. Scale bar = 10μm. B1R: Bradykinin 1 receptor; B2R: Bradykinin 2 receptor; STS: Staurosporine. Blue: DAPI (4´,6-diamidino-2-phenylindole), Red: B1R pAb (upper panel) or B2R pAb (lower panel) with Alexa-Fluor 488-conjugated goat anti-mouse IgG.

Immunofluorescence analysis of PC12 cells were analyzed for B1R and B2R expression at 3h, 12h and 24h in response to STS treatment. In particular B1R expression was not detected until 12h post-STS treatment (Fig 1a). Conversely, B2R, which was expressed in the controlled cells, showed to be highly upregulated 3h post-STS treatment (Fig 1b).

### B2R inhibition prevents intracellular calcium release post-STS treatment

**Fig 2:**
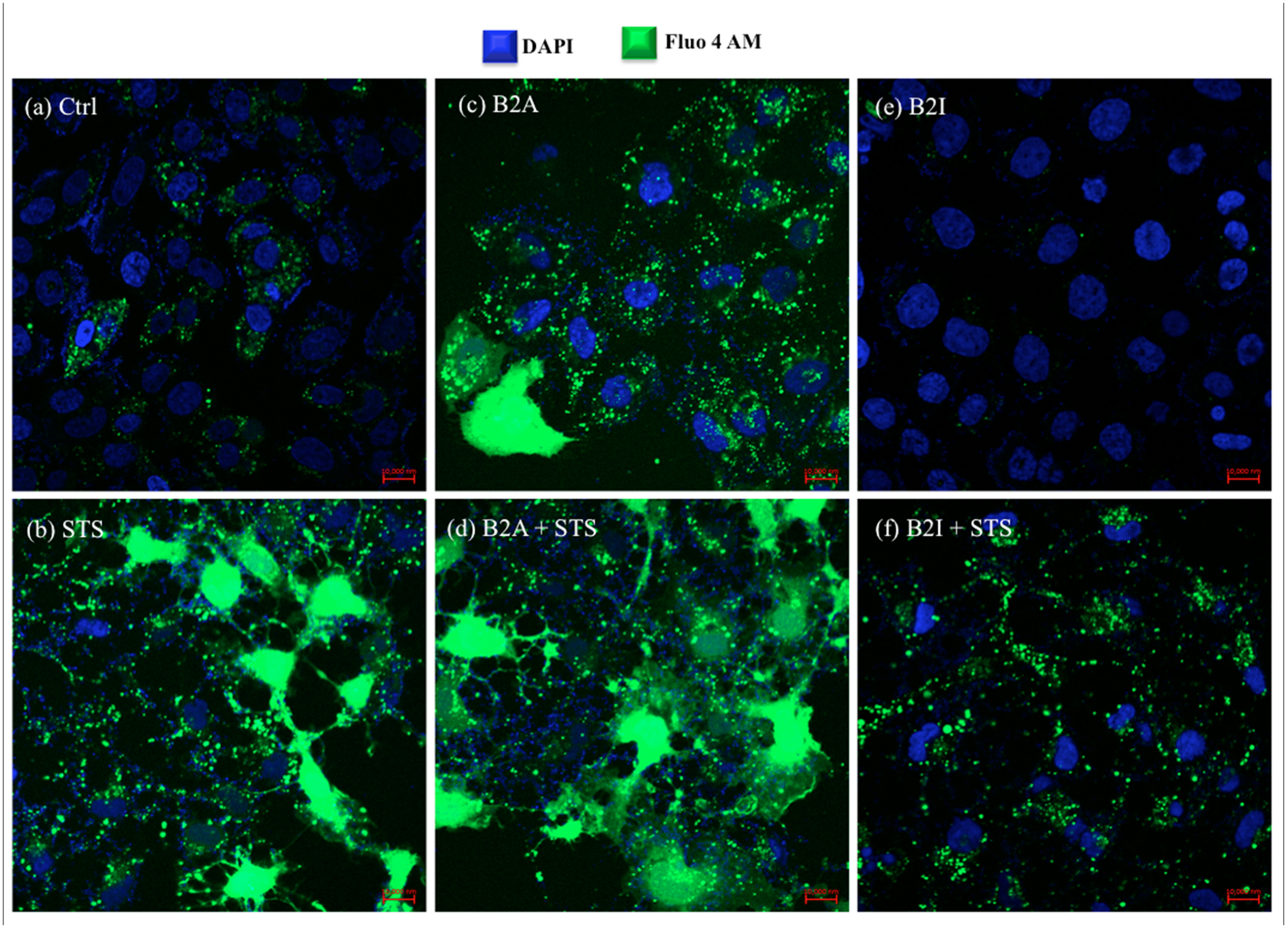
Fluo-4 AM staining of PC12 cells 3h post-STS treatment depicting intracellular calcium release. Immunofluorescent images of **(a)** PC12 cells control, **(b)** STS treated, **(c)** B2A pre-treated, **(d)** B2A pre-treated and STS treated, **(e)** B2I pre-treated, and **(f)** B2I pre-treated and STS treated. Scale bar = 10 000nm. STS: Staurosporine; B2A: Bradykinin 2 Receptor activated; B2I: Bradykinin 2 Receptor inhibited.

To assess the contribution of STS treatment on calcium release and its effect on B2R, PC12 cells were stained with a Fluo-4 AM agent. STS treatment actually showed increased intracellular calcium level 3h post-treatment when compared to the control (Fig 2a and 2b). Likewise, calcium release was observed in B2R agonist (B2A) pre-treated PC12 cells and the effect was exacerbated at the time point of 3h post-STS treatment (Fig 2c and 2d). However, calcium release was significantly inhibited in B2R inhibitor (B2I) pre-treated PC12 cells and even 3h post-STS treatment (Fig 2e and 2f). Data observed from STS treatment indicate that STS-induced calcium release is actually B2R-dependent and its inhibition blocks the release even in the presence of STS neurotoxicity. As for B1R coupled with STS treatment, calcium release was not as statistically significant as observed in B2R (data not shown).

### B2R transcriptional expression increases 3h post-STS treatment

**Fig 3:**
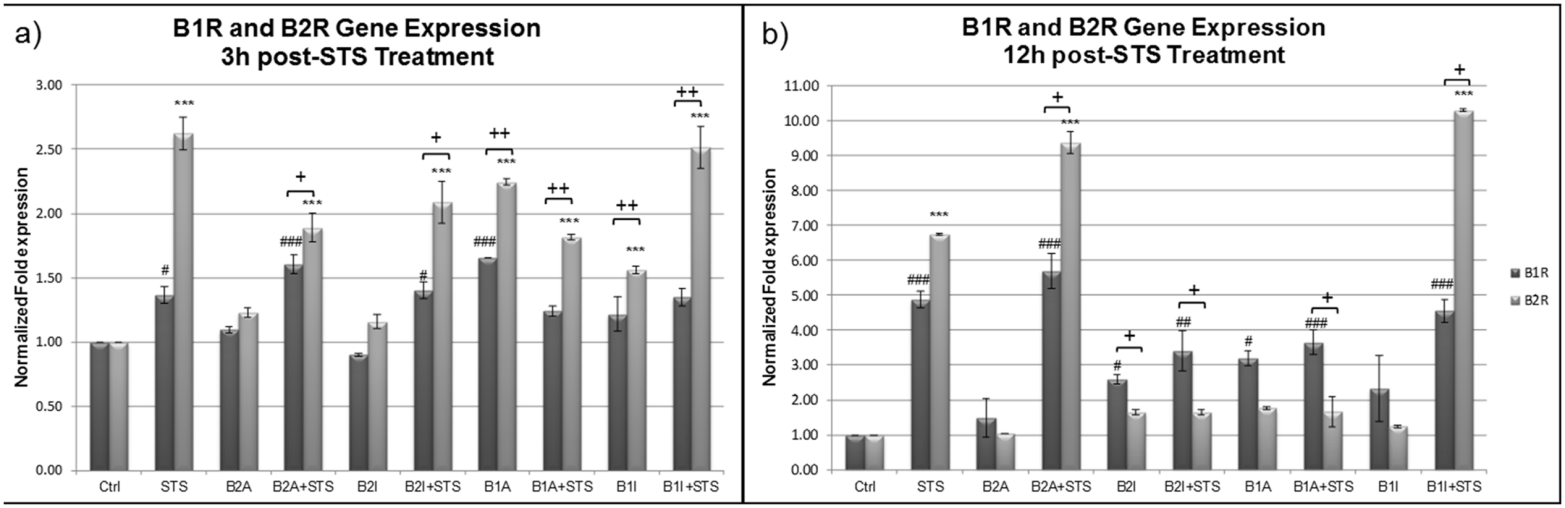
Expression of the B1R and B2R genes in PC12 cells (a) 3h and (b) 12h post-STS treatment, using RT-PCR assay. (n = 3, triplicate). Each gene was normalized to the housekeeping gene GAPDH, and the control was used as a reference for comparative analysis. Error bars represent standard errors of the mean. Symbols indicate statistical difference: B1R vs B1R-Ctrl, (###) p<0.001, (##) p<0.01, (#) p<0.05; B2R vs B2R-Ctrl, (***) p<0.001 (one-way ANOVA with Bonferroni correction for multiple comparisons). B1R vs. B2R, (++) p<0.1, (+) p<0.05 (Paired t test).

Since B1R and B2R were shown to be involved in STS mediated apoptotic cell death of PC12 cells, but each one at a different time point and with a different dynamics, we studied the expression level of these receptors at the molecular level assessing their mRNA expression. RT-PCR was performed on PC12 cells 3h and 12h post STS treatment to quantify the levels of B1R and B2R gene expression. Fig 3a shows that at 3h post-STS treatment B2R expression is highly induced compared to B1R in almost all conditions. Noteworthy, B2R gene expression is the highest following STS treatment and upon inactivation of B1R followed by STS treatment, suggesting a compensatory role of B2R when B1R is inactivated. In addition, an exaggerated increase in B1R and B2R gene expression is observed after 12h of STS treatment (Fig 3b) with B1R gene expression higher than B2R expression in almost all conditions. Only in 3 conditions, namely, STS, B2A+STS and B1I+STS, B2R expression remained higher than B1R expression.

### B2R translational expression increases 3h post-STS treatment

**Fig 4:**
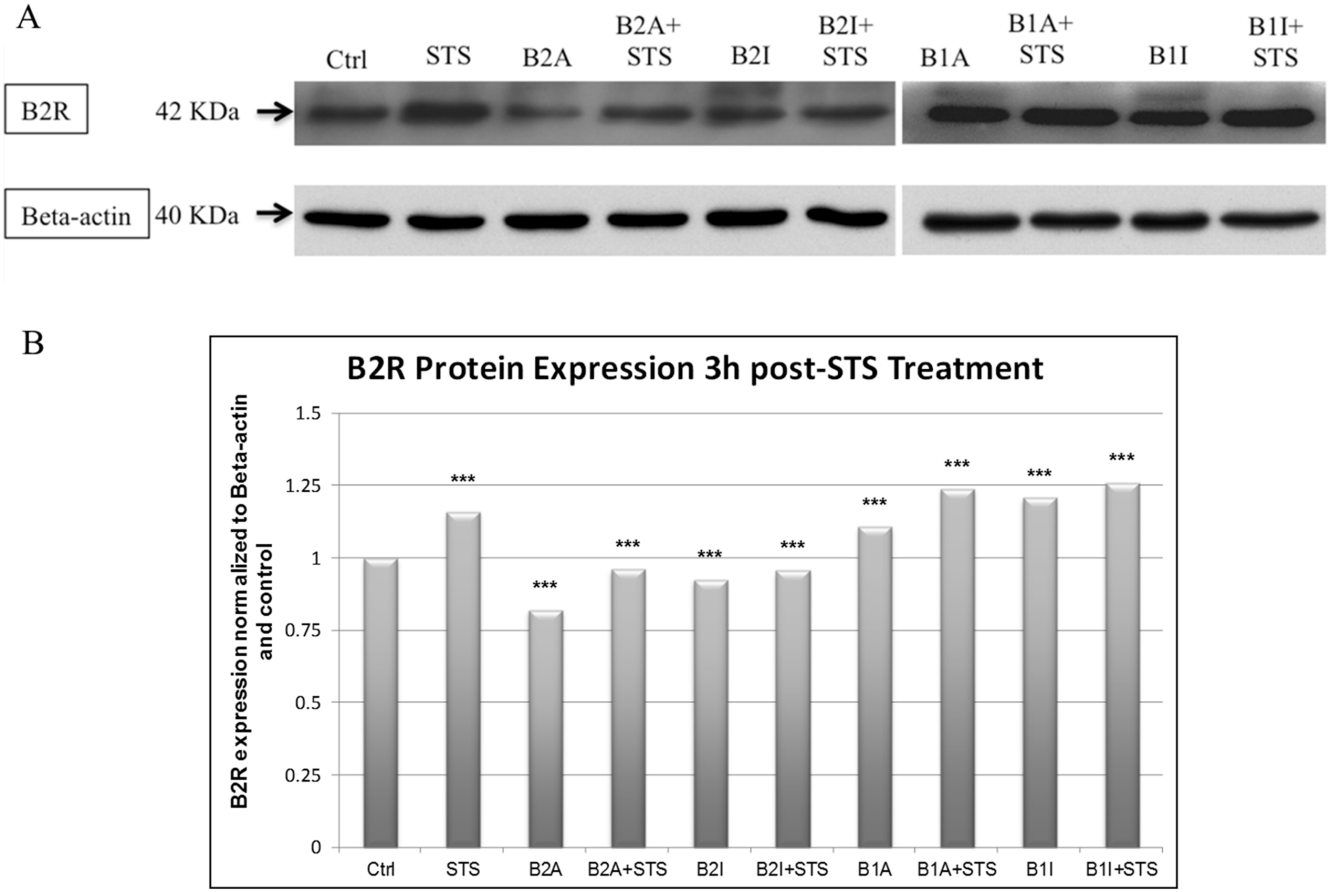
B2R protein expression upon 3h STS treatment of PC12 cells. **(a)** Western Blot of protein extracts from PC12 cells. **(b)** Histogram representing densitometry analysis of B2R expression using Image J software (n = 3). Error bars represent standard errors of the mean. Symbols indicate statistical difference: B2R vs. B2R-Ctrl, (***) p<0.001 (one-way ANOVA with Bonferroni correction for multiple comparisons).

Since our results (Figs 1 and 2) indicated that B2R is the major KKS player involved at early time points after STS mediated apoptosis, B2R protein expression was studied in PC12 cells 3h post-STS treatment. Our data revealed an increase in B2R protein expression in STS-treated PC12 cells and in B1A, B1A+STS, B1I, B1I+STS conditions (Fig 4).

### Neurosystems Biology Study

**Fig 5:**
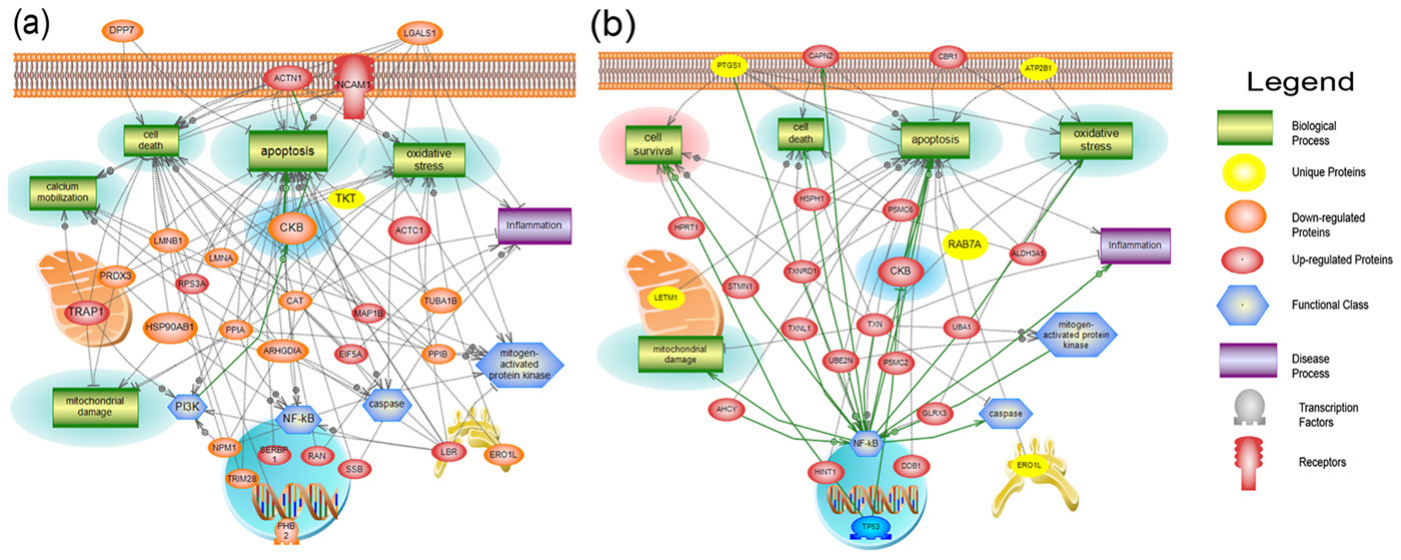
Pathways study of STS-induced altered proteins. Global Interaction Proteome of **(a)** STS treated cells and **(b)** B2I+STS treated cells. The green rectangles, violet rectangles and blue hexagons are reflective of biological processes, disease processes and functional classes, respectively. The different colors of proteins reflect their alteration: upregulated (red), downregulated (orange), downregulated and unique to the treated group (yellow). The green highlights represent the detrimental effects observed following STS or B2I+STS treatment and the pink highlight represents the protective effect observed following B2I+STS treatment. STS: Staurosporine, B2I: Bradykinin 2 receptor inhibited. Creatine kinase B-type [15], designated by blue highlight is shown to be upregulated in the STS+ B2I treatment where cell survival pathway was detected.

The software Pathway Studio v.10 (2015) was used to search for possible protein-protein interactions, common regulators, cell processes, and related pathways for associations with STS—induced altered proteins before and after treatment with B2I. The network was generated using Shortest Path algorithm to map interactions between altered proteins and only the identified proteins with 2 or more references were kept. Fig 5a depicts the global interaction proteome of the STS group while Fig 5b reflects the global interaction proteome of the B2I+STS group. Through this approach, we found that proteins belonging to different structural and functional families were involved in processes such as mitochondrial damage, cell death, inflammation, oxidation, and apoptosis. Nevertheless, the most important discovery was that, in the B2I+STS cohort, fewer proteins were involved in these detrimental processes and new proteins appeared to be involved in cell survival, namely, Thioredoxin (TXN), Carbonyl reductase 1 (CBR1), aldehyde dehydrogenase 3 family, member A1 (ALDH3A1), Histidine triad nucleotide-binding protein 1 (HINT1) and Microtubule-associated protein RP/EB family member 1 (MAPRE1). Moreover, NF-κB was the only functional class that contained more hits in the B2I+STS group than in the STS group. Among the differential proteins identified, creatine kinase B-type [15] was shown to be altered in both treatment STS and STS+B2I (Fig 5a and 5b). The MSMS spectra of three unique peptides are shown for creatine kinase B-type (S1 Fig).

## Discussion

Neuroinflammation resulting from TBI represents a major target for neurotherapy. As stated in a recent review, many studies were conducted on the well-known KKS inflammatory system in order to tackle the role of B1R and B2R in TBI [13]. Although encouraging, the results failed to determine the exact contribution (protective or detrimental) of the B1R and B2R post-TBI. Thus, our study aimed at verifying the involvement of the B1R and B2R, and demonstrating the major role of the B2R in PC12 cells post STS-induced apoptotic cell death.

Of interest, we were able to identify the early contribution of the B2R in STS-treated PC12 cells, as compared to the B1R (Fig 1a and 1b). We have shown that STS significantly inhibits proliferation and induces cell death as early as 3h post-treatment. After 24h post STS treatment, a significant decrease in cell survival (approximately 90%) was detected (S2 Fig). Such treatment resulted in a differential dynamics of B2R and B1R expression, as shown in Fig 1a and 1b, and as illustrated in the MTT cytotoxicity assay (S2 Fig), where the proliferation activity of PC12 cells was found to be inversely correlated with STS in the culture medium. Indeed, the constitutive B2R was highly induced 3h post-STS treatment, while B1R induction started 12h post-STS treatment. This result can be explained by the fact that B1R is localized in the endoplasmic reticulum (ER) and exists with a very low expression on the plasma membrane; in contrast, B2R is capable of efficiently exiting the ER, targeting the plasma membrane [9]. Furthermore, this differential expression of B1R versus B2R may be attributed to the presence of p53 Responsive Element in the promoter region of B2R gene. Thus, upon activation/over-expression of p53 post STS treatment (apoptotic stimulus), this p53 Responsive Element led to activation and enhanced expression of B2R [29, 30].

In addition, our RT-PCR results confirmed a high B2R expression compared to B1R at 3h post-STS treatment in PC12 cells and an even higher B1R expression compared to B2R at 12h post-STS treatment (Fig 3). Notably, a similar trend was observed between protein expression and mRNA levels of B2R in STS treated cells, with the highest expression found in STS, B1A+STS, and B1I+STS conditions (Fig 4). Taken together, these results suggest a crosstalk between the B1R and B2R and the main participation of the B2R.

**Fig 6:**
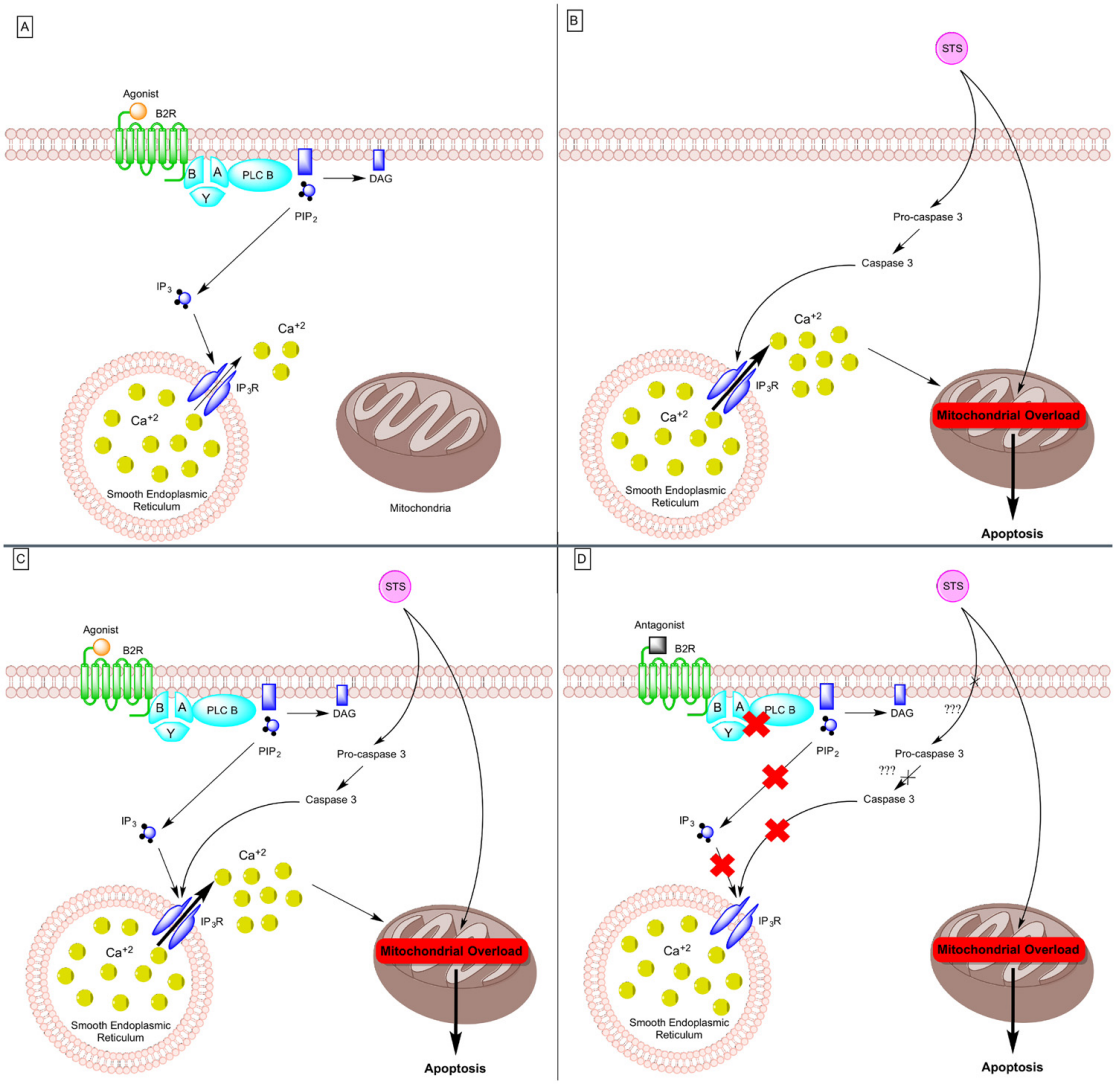
Effects of STS and B2R antagonist on calcium release and apoptosis. **(a) B2R activated**. Once B2R, a GPCR, is activated, the Gα subunits detaches from the Gβ/Gγ subunits, leading to activation of PLCb, which will trigger the conversion of PIP2 into DAG and IP3. IP3 will then activate IP3R on sER, leading to the release of calcium in the cytosol. **(b) STS treatment**. STS activates the cytosolic pro-caspase 3 protein, which is converted into caspase 3 and acts on IP3R, leading to a high amount of calcium release. This high amount of calcium will result in mitochondrial overload, initiating the apoptotic cascade. **(c) B2R activated and STS treatment**. This is a combination of the STS and B2R activation effects, with a high amount of calcium being released in the cytosol, followed by mitochondrial overload and apoptosis. **(d) B2R inactivated and STS treatment**. Once B2R is inactivated, no calcium is being released, which can be explained by the fact that IP3 is no more produced and/or that caspase 3 is not acting on IP3R (but we still don’t know how). However, the apoptotic cascade is still active, suggesting that another pathway exists for STS to exert its apoptotic effect. STS: Staurosporine; GPCR:G-protein-couples receptor; PLCb: Phospholipase C beta; PIP2: Phosphatidyinositol 4,5 biphosphate; DAG: diglyceride; IP3: inositol 1,4,5 triphosphate; IP3R: IP3 receptor; sER: smooth endoplastic reticulum.

An interesting observation was the ability of the B2R to prevent intracellular calcium release once inhibited, even following STS treatment (Fig 2). By combining our results with previous studies, we were able to draw the following potential B2R mechanism. Under normal circumstances and following activation of the B2R, Ca^2+^ channels located on the surface of the smooth ER are most probably opened via binding of inositol 1,4,5-trisphosphate (IP_3_), a downstream product of GPCRs [31], leading to calcium release in the cytoplasm [32]. Otherwise, STS leads to the cleavage of the cytosolic pro-caspase 3 into caspase 3, which binds and induces a conformational change of IP_3_ receptor, exacerbating intracellular Ca^2+^ release [33]. This high calcium concentration is sequestered by the mitochondria [34]. Mitochondrial calcium overload induces the opening of permeability transition pore, mitochondrial dysfunction and apoptosis [35]. Furthermore, since we demonstrated that inhibition of B2R impedes intracellular calcium release in STS-treated PC12 cells (Fig 2), it can be suggested that following inactivation of B2R, an intermediate player in the downstream mechanism of the B2R and STS is getting activated. This intermediate participant can either prevent the activation of caspase 3 or the binding of caspase 3 to IP_3_ receptor, shutting down intracellular calcium release. Nonetheless, apoptosis is still observed in all STS conditions. Therefore, alleviating intracellular calcium release by inhibiting B2R is not sufficient to prevent STS-induced apoptosis. This whole mechanism has been summarized in Fig 6.

Finally, a shotgun neuroproteomics platform was utilized to study the global dynamics of proteome changes post-STS treatment and compare it to B2I treatment. Specifically, an *in vitro* LC-MS/MS proteomic analysis of neuronal cells subjected to STS-induced apoptosis, which mimics intracellular fluid in the central nervous system post-TBI [36], was used to highlight protein markers related to brain insult [37, 38]. Our experimental approach, although similar to the approach of Guingab-Cagmat et al., was more focused on the KKS, especially on the inhibition of the B2R [36]. Our proteomics results revealed that inhibition of the B2R and activation of the transcription factor NF-κB are essential for the survival of PC12 cells but not sufficient to completely prevent STS-mediated apoptosis. Therefore, future studies should focus on the “survival” promoting proteins identified in the downstream events of the B2R: TXN, CBR1, ALDH3A1, HINT1 and MAPRE1 (Fig 5a and 5b). These proteins are important as they indicate the mechanism of how PC12 cells may resist STS-mediated apoptosis when B2R is inhibited and can have a protective role against TBI. Several of these proteins have been linked to cell death pathways as well as inflammatory mechanisms as shown in previous TBI-related studies [7, 36, 39, 40]. A detailed depiction of the exact role of these proteins pertaining to the designated altered pathways is shown in S1 and S2 Tables. Moreover, this small number of identified proteins makes them more suitable and convenient for further experiments. In addition, among the identified proteins, the brain specific creatine kinase B-type [15] was found to be upregulated in the STS+ B2I treatment. CKB has been linked to neuroprotection via reducing oxidative stress as shown in a study performed on rat striatal slices [41].

To sum up, the fact that inhibition of B2R is impeding intracellular calcium release following STS treatment suggests a potential therapeutic role of the B2R. In addition, by providing the cell with a “survival” capacity, B2R antagonist is a legitimate therapeutic tool candidate, especially to treat diseases where apoptosis and excessive intracellular calcium release are the main players, such as Alzheimer’s disease [42] and Huntington’ disease [43]. Although PC12 cells represent a simple and convenient *in vitro* neuronal model to study the inflammatory response of TBI, one must keep in mind that, as with all models, PC12 cells have their limitations. Any result obtained with this cell line does not assure that the same findings will be found in *in vivo* models. Consequently, any suggested hypothesis driven from PC12 cells should also be tested in primary neuronal cell cultures and *in vivo* models, as well as in TBI itself.

## Conclusions

In conclusion, our study demonstrated that STS-induced apoptosis in PC12 cells involves, primarily, the contribution of B2R, and is mediated via calcium signaling and apoptotic pathways. These data were substantiated with neuroproteomics data.

## Supporting Information

S1 Fig MS/MS spectra creatine kinase B-type (P07335) brain specific protein. Identified MS/MS spectra of three unique peptides corresponding to creatine kinase B-type (P07335) brain specific protein shown to be downregulated in the STS treatment and upregulated in the STS + B2I treatment (please refer to Fig 5). The three peptides shown are: (A) Peptide DLFDPIEDR, (B) Peptide FCTGLTQIETLFK and (C) Peptide GTGGVDTAAVGGVFDVSNADR.
(TIF)

S2 Fig Inhibition in the proliferative activity of PC12 cells in response to STS treatments after 3h, 12h and 24h using MTT assay. Illustrated is an MTT cytotoxicity assay to assess the proliferation activity of PC12 cells. It is observed that after 3h of treatment, STS is killing approximately 60% of PC12 cells and on average 50% of pre-treated PC12 cells. Nevertheless, the potent role of STS is highlighted by the fact that at this same time point, B1R and B2R agonists and antagonists are showing an increase in the proliferative activity of PC12 cells but fail to protect PC12 cells from the harmful effect of STS. Although B1A, B1I, B2A and B2I still increase the mitochondrial enzymatic activity of PC12 cells at 12h and 24h post- treatment, they do not exhibit any protection against STS treatment that kills 90% of PC12 cells. It is also important to note that activation or inhibition of B1R and B2R are showing similar results, at all time points: increase in proliferative activity of PC12 cells before STS treatment, and decrease in proliferative activity of PC12 cells following STS treatment. Results are expressed as a percentage of the studied group compared to its control. The data are reported as mean +/- SD (n = 3, triplicate, *P<0.05, **P<0.01, ***P<0.001).
(TIF)

S1 Table Altered Proteins and Molecular function/Biological Process Relations in PC12 post-STS treatment. The table states the proteins’ names and depicts the exact relation of the altered proteins pertaining to the designated altered molecular function and biological Process.
(XLSX)

S2 Table Altered Proteins and Molecular function/Biological Process Relations in PC12 post STS and B2R inhibitor treatment. The table states the proteins’ names and depicts the exact relation of the altered proteins pertaining to the designated altered molecular function and biological Process.
(XLSX)
