# Correction: Characterization of the Kallikrein-Kinin System Post Chemical Neuronal Injury: An *In Vitro* Biochemical and Neuroproteomics Assessment

**DOI:** 10.1371/journal.pone.0136345

**Published:** 2015-08-19

**Authors:** Amaly Nokkari, Tarek H. Mouhieddine, Muhieddine M. Itani, Wassim Abou-Kheir, Georges Daoud, Rui Zhu, Yehia Mechref, Jihane Soueid, Moustafa Al Hariri, Stefania Mondello, Ayad A. Jaffa, Firas Kobeissy

The seventh author’s name is spelled incorrectly. The correct name is: Yehia Mechref.
