# Supplementary figures and images for: Characterization of the Kallikrein-Kinin System Post Chemical Neuronal Injury: An In Vitro Biochemical and Neuroproteomics Assessment
Source: PLoS One. 2015 Jun 5;10(6):e0128601. doi: 10.1371/journal.pone.0128601 (PMC4457722; doi:10.1371/journal.pone.0128601)

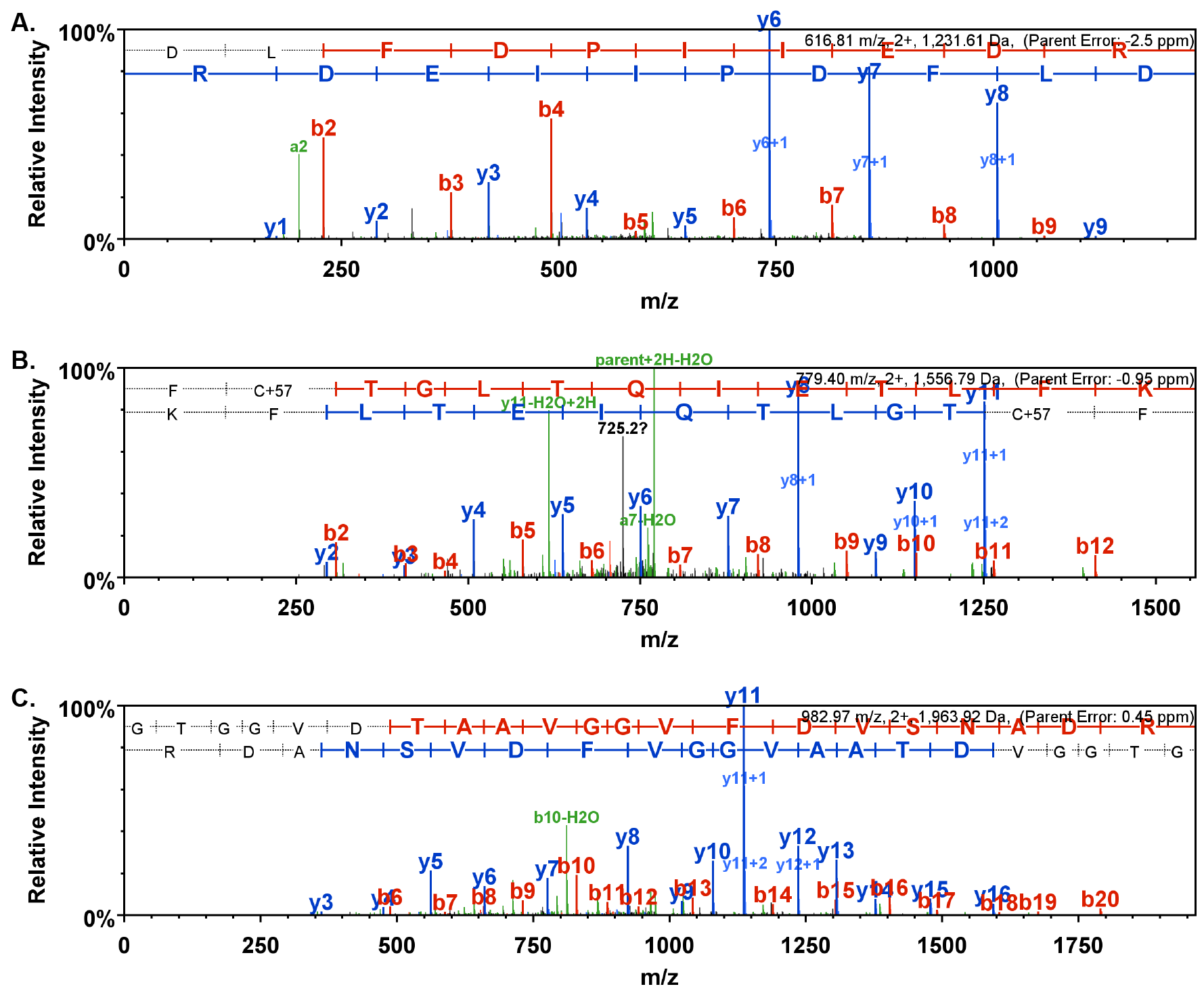

Supplement: S1 Fig — Identified MS/MS spectra of three unique peptides corresponding to creatine kinase B-type (P07335) brain specific protein shown to be downregulated in the STS treatment and upregulated in the STS + B2I treatment (please refer to Fig 5). The three peptides shown are: (A) Peptide DLFDPIEDR, (B) Peptide FCTGLTQIETLFK and (C) Peptide GTGGVDTAAVGGVFDVSNADR. (TIF) [file pone.0128601.s001.tif]

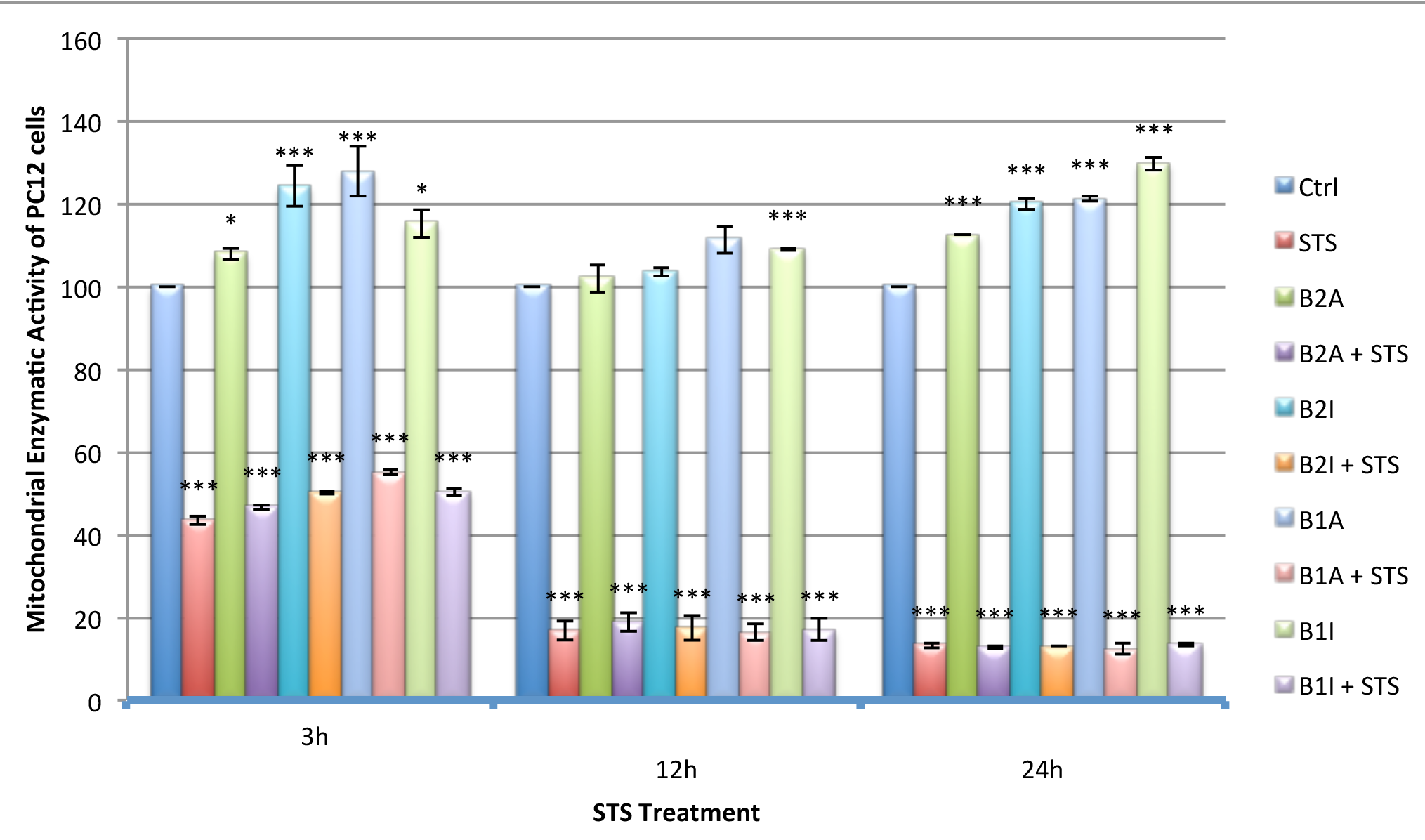

Supplement: S2 Fig — Illustrated is an MTT cytotoxicity assay to assess the proliferation activity of PC12 cells. It is observed that after 3h of treatment, STS is killing approximately 60% of PC12 cells and on average 50% of pre-treated PC12 cells. Nevertheless, the potent role of STS is highlighted by the fact that at this same time point, B1R and B2R agonists and antagonists are showing an increase in the proliferative activity of PC12 cells but fail to protect PC12 cells from the harmful effect of STS. Although B1A, B1I, B2A and B2I still increase the mitochondrial enzymatic activity of PC12 cells at 12h and 24h post- treatment, they do not exhibit any protection against STS treatment that kills 90% of PC12 cells. It is also important to note that activation or inhibition of B1R and B2R are showing similar results, at all time points: increase in proliferative activity of PC12 cells before STS treatment, and decrease in proliferative activity of PC12 cells following STS treatment. Results are expressed as a percentage of the studied group compared to its control. The data are reported as mean +/- SD (n = 3, triplicate, *P<0.05, **P<0.01, ***P<0.001). (TIF) [file pone.0128601.s002.tif]
